# Mechano-adaptive sensory mechanism of α-catenin under tension

**DOI:** 10.1038/srep24878

**Published:** 2016-04-25

**Authors:** Koichiro Maki, Sung-Woong Han, Yoshinori Hirano, Shigenobu Yonemura, Toshio Hakoshima, Taiji Adachi

**Affiliations:** 1 Department of Biomechanics, Institute for Frontier Medical Sciences, Kyoto University, 53 Shogoin-Kawahara-cho, Sakyo, Kyoto 606-8507, Japan; 2 Department of Micro Engineering, Graduate School of Engineering, Kyoto University, Yoshida Honmachi, Sakyo, Kyoto 606-8501, Japan; 3 National Institute for Nanomaterials Technology, Pohang University of Science and Technology, 77 Cheongam-ro, Nam-Gu, Pohang, Gyeongbuk 790-784, Korea; 4 Structural Biology Laboratory, Graduate School of Biological Sciences, Nara Institute of Science and Technology, 8916-5 Takayama, Ikoma, Nara 630-0192, Japan; 5 Ultrastructural Research Team, RIKEN Center for Life Science Technologies, 2-2-3 Minatojima-minamimachi, Chuo-ku, Kobe, Hyogo 650-0047, Japan

## Abstract

The contractile forces in individual cells drive the tissue processes, such as morphogenesis and wound healing, and maintain tissue integrity. In these processes, α-catenin molecule acts as a tension sensor at cadherin-based adherens junctions (AJs), accelerating the positive feedback of intercellular tension. Under tension, α-catenin is activated to recruit vinculin, which recruits actin filaments to AJs. In this study, we revealed how α-catenin retains its activated state while avoiding unfolding under tension. Using single-molecule force spectroscopy employing atomic force microscopy (AFM), we found that mechanically activated α-catenin fragment had higher mechanical stability than a non-activated fragment. The results of our experiments using mutated and segmented fragments showed that the key intramolecular interactions acted as a conformational switch. We also found that the conformation of α-catenin was reinforced by vinculin binding. We demonstrate that α-catenin adaptively changes its conformation under tension to a stable intermediate state, binds to vinculin, and finally settles into a more stable state reinforced by vinculin binding. Our data suggest that the plastic characteristics of α-catenin, revealed in response to both mechanical and biochemical cues, enable the functional-structural dynamics at the cellular and tissue levels.

A combination of contractile forces in individual cells drives tissue dynamics such as morphogenesis^1^,^2^,^3^ and wound healing^4^. The cadherin-based adherens junctions (AJs) function as direct links between the contractile actomyosin cytoskeletons of different cells^5^,^6^. AJs balance the intercellular tensions by the adaptive assembly of the cytoskeletal actin filaments^7^,^8^,^9^,^10^. In this mechano-adaptive mechanism, α-catenin, a tension-sensing component of AJs, is critical regulator of vinculin binding^11^,^12^,^13^, which recruits another actin filament to AJ^14^,^15^.

The molecular mechanisms of the regulation of vinculin binding by α-catenin have been investigated using various approaches. The molecular and cellular study of Yonemura *et al.*^16^ has revealed that α-catenin under intercellular tension exposes the cryptic vinculin binding site (VBS). The process is triggered by the release of the autoinhibited conformation caused by an intramolecular interaction between its M_I_ domain (residues 275–391) containing VBS (residues 325–360) and the vinculin-inhibitory M_II_–M_III_ domain (residues 510–697). The autoinhibited conformation has been determined in the crystallized α-catenin [PDB code: 4K1N]^17^, where the vinculin-binding surface of VBS is buried in the helix bundle of M_I_ domain that is structurally stabilized by M_II_–M_III_ helix bundles. Single-molecule experiments using magnetic tweezers have suggested that the disruption of M_I_/M_II_–M_III_ interaction requires only approximately 5-pN tension^18^, which is close to the range of forces generated by a single myosin molecule^19^. Thereby, the M_I_/M_II_–M_III_ interaction holds the key to the mechanical activation of α-catenin, recruiting vinculin under tension.

However, α-catenin mechanically activated under tension faces a critical problem common to all tension-sensing proteins. Usually, an external force acts as a biomolecule denaturant^20^,^21^. A study^22^ using atomic force microscopy (AFM) has reported a complete unfolding of a protein in approximately 30 s under low tension of approximately 13 pN. Furthermore, α-helical proteins such as α-catenin have lower mechanical stabilities than other β-sheet proteins^23^. During the tension-sensing process, the helical conformation of VBS should be conserved to associate with vinculin^24^. Thus, the most significant question is how does α-catenin, one of the tension-sensing proteins, retain its activated state while avoiding the successive unfolding under denaturing tension.

Here, we revealed that α-catenin under tension changed its conformation to a stable intermediate state. We investigated the mechanical behaviors of mutated, segmented, and vinculin-bound α-catenin using single-molecule force spectroscopy (SMFS)^25^,^26^,^27^ employing AFM. To reveal the conformational changes in α-catenin under tension, we adopted the following two types of loading conditions: (a) direct loading without mechanical activation and (b) loading with a holding period to wait for mechanical activation.

## Results

### Mechano-adaptive conformational change in M_I_-M_III_

**Figure 1:**
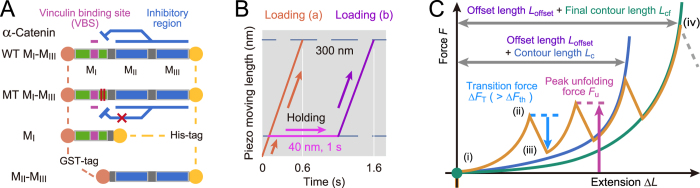
SMFS using AFM. (**A**) α-Catenin fragments: WT M_I_-M_III_ (residues 276–634), mutant (MT) M_I_-M_III_ (M319G and R326E), M_I_ (residues 276–393), and M_II_–M_III_ (residues 385–634). (**B)** Loading conditions (a) and (b). Single α-catenin molecules were loaded by NanoWizard 3 BioAFM (JPK Instruments, Berlin, Germany). The piezo-actuator was moved upward by 300 nm at a constant speed of 500 nm/s (“Loading (a)” in this study, orange line). To analyze the mechanical behavior of WT M_I_-M_III_ after mechanical activation, we introduced a holding time of 1 s at 40 nm of constant piezo-moving length (“Loading (b),” orange (initial loading), magenta (holding), and purple (further loading)). (**C**) Force curve analysis. First, the force curves with saw-tooth patterns (state (i) to (iv), orange curve) caused by single-molecule behaviors were identified, with the rupture force and final stiffness in the last peak (state (iv)). Next, the offset length *L*_offset_ was determined by WLC-model fitting to the last peak (green curve) with the fixed final contour length *L*_cf_ (143.2 nm (WT/MT M_I_-M_III_), 46.8 nm (M_I_), and 99.6 nm (M_II_–M_III_)). Finally, we measured the contour length *L*_c_ and peak unfolding force *F*_u_ at each force peak with transition force Δ*F*_T_ (state (ii) to (iii)) greater than the threshold Δ*F*_th_ (Methods: “Force curve analysis”).

We used four types of α-catenin fragments (Fig. 1A): wild type (WT) M_I_-M_III_ (residues 276–634), mutant (MT) M_I_-M_III_ (M319G and R326E), M_I_ (residues 276–393), and M_II_–M_III_ (residues 385–634). The mutant fragment, in which the autoinhibiting MI/M_II_–M_III_ interaction is disrupted, behaved as an activated α-catenin that interacts with vinculin under no tension (Supplementary Figure 1). The weakened M_I_/M_II_–M_III_ interaction causes a change in the angle between helix bundles M_II_ and M_III_^17^ and the destabilization of helix bundle M_I_^28^. α-Catenin fragments bound to the glass substrate at the C-terminus exploiting NTA-Ni^2+^-His_6_ affinity were loaded at the N-terminus using GST-GSH affinity. The autoinhibitory WT M_I_-M_III_ fragment was examined in the following two types of loading conditions (Fig. 1B): (a) direct loading (orange line) and (b) loading with a holding period (magenta line) to wait for mechanical activation. The force curves corresponding to the single α-catenin molecules were extracted and their characteristics were analyzed (Fig. 1C); we calculated contour length *L*_c_ to identify intermediate states during unfolding and the measured peak unfolding force *F*_u_ to estimate the mechanical stabilities of the abovementioned α-catenin fragments.

**Figure 2:**
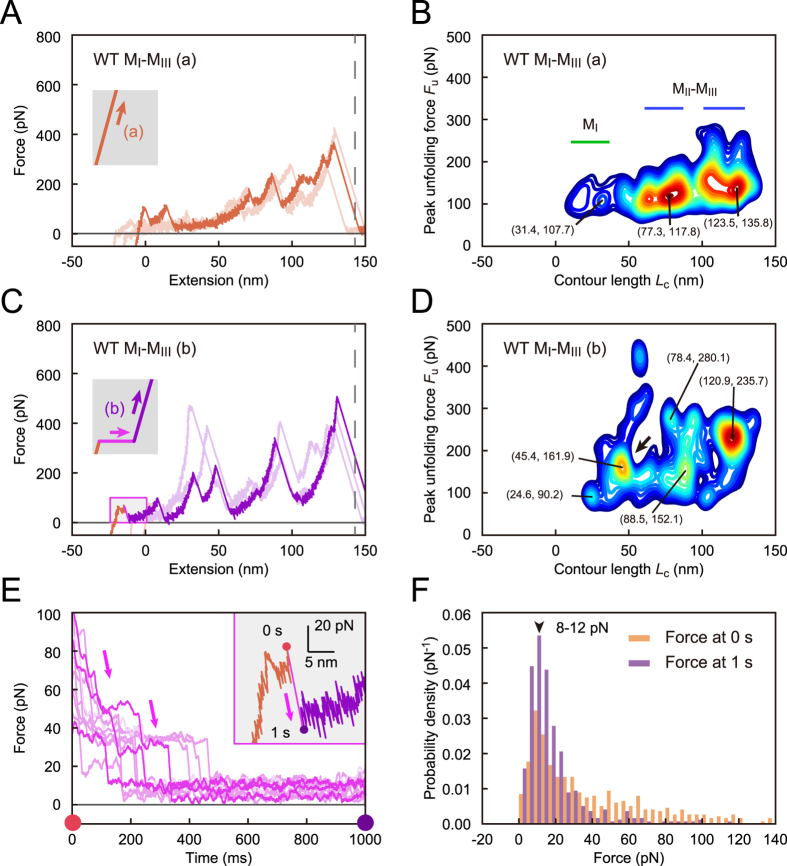
Conformational change in M_I_-M_III_ domain under tension. (**A**) Force curves for WT M_I_-M_III_ fragment in loading (a). The force curves are shifted to the left by the offset length *L*_offset_. (**B**) Contour map of number density *n*_d_ of force peaks in loading (a) based on 797 force curves. Color contours are set from the maximum value of *n*_d_ (red) to 0.4 × the value (blue). (**C**) Force curves in loading (b). (**D**) Contour map in loading (b) based on 514 force curves. (**E**) Force relaxation curve during the holding time. (**F**) The probability density of force at 0 s (orange bars) and 1 s (purple bars) during the holding time.

WT M_I_-M_III_ fragment in loading (a) unfolded each domain under tension. To analyze the stochastic unfolding trajectories from obtained force curves (Fig. 2A), we evaluated the number density of force peaks, *n*_d_ (*L*_c_, *F*_u_) on the basis of scatter plots of *F*_u_ versus *L*_c_. The contour map of the number density *n*_d_ showed three wide horizontal regions with similar intervals (Fig. 2B; the contour map with regard to net extension is shown in Supplementary Figure 2A). The low *n*_d_ in the initial region (green line) indicated that the weak helix bundle of M_I_ unfolded easily, with infrequent force peaks. However, the last two regions (blue lines) with high *n*_d_, i.e., with frequent force peaks, corresponded to two stable helix bundles of M_II_–M_III_ domain. These results indicated that M_I_-M_III_ domain unfolding depended on the mechanical stability of their helix bundles under direct loading.

However, WT M_I_-M_III_ in loading (b) showed force peaks within a rather greater force range (Fig. 2C), and the contour map of *n*_d_ showed greater peak unfolding force *F*_u_ (Fig. 2D) than those in loading (a) (Fig. 2B). This result indicated that the conformation of M_I_-M_III_ domain changed to another intermediate state that required higher tension for further unfolding. As the peak unfolding force *F*_u_ increased along the entire contour length *L*_c_ (Fig. 2D), the conformational change must have occurred at all three M_I_-M_III_ domains. The contour map showed a specific peak at *L*_c_ of approximately 45.4 nm (arrow, Fig. 2D); the net extension was approximately 19.0 nm (Supplementary Figure 2B), <40% of completely extended length of M_I_ domain. This observation suggested that VBS in M_I_ domain was conserved in this region. During the holding time, the force relaxation curve showed stepwise relaxations (Fig. 2E), implying dynamic transitions in the M_I_-M_III_ conformation. The force distribution at the beginning (0 s) of the holding time settled into a narrow distribution approximately 10 pN at the end of this period (1 s, Fig. 2F), showing that the M_I_-M_III_ conformation finally equilibrated under low tension. We suggest that M_I_/M_II_–M_III_ interaction was partly diminished during the holding time; the average extension of approximately 2.8 nm in the holding period was longer than the reported extension^28^ that was approximately 1.0 nm for the mechanical activation of M_I_-M_III_ domain. The probability density of the force peaks in loading (b) in the initial extension was lower than that in loading (a), which supported this suggestion (Supplementary Figure 2C). Thus, we revealed that M_I_-M_III_ domain of α-catenin adaptively changes the conformation to another stable state under low tension, with weakened M_I_/M_II_–M_III_ interaction.

### M_I_/M_II_–M_III_ interaction as a conformational switch

**Figure 3:**
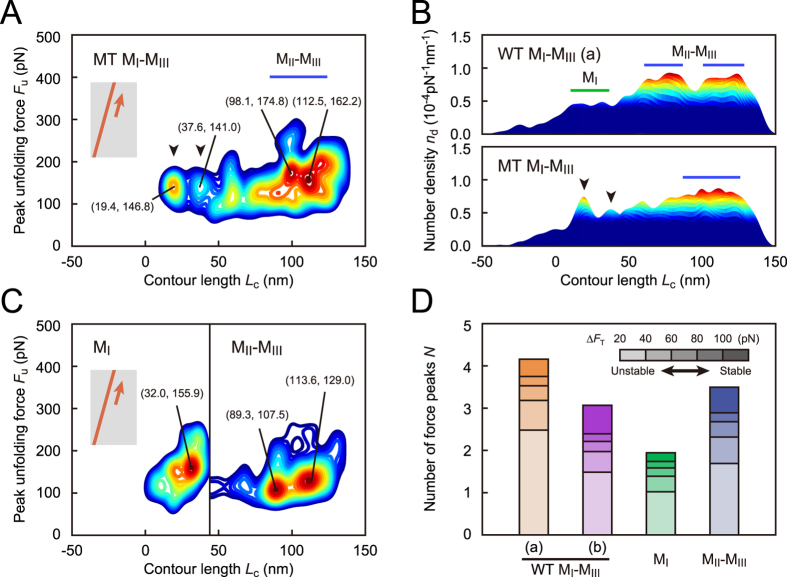
Mechanical behaviors of mutated and segmented α-catenin. (**A**) Contour map for MT M_I_-M_III_ fragment based on 576 force curves. (**B**) Comparison of the number density *n*_d_ in contour maps from a side view in WT and MT M_I_-M_III_ fragments. (**C**) Contour maps for M_I_ and M_II_–M_III_ fragments based on 181 and 785 force curves, respectively. The contour map for M_II_–M_III_ fragment is shifted to the right by 46.8 nm, which corresponds to the fully-extended length of M_I_ domain. (**D**) The average number of force peaks *N* per curve. The stacked bar graphs display *N* values, with color intensity illustrating the range of transition force Δ*F*_T_. The substructure stability increases with the increasing Δ*F*_T_ values.

To elucidate the conformational changes in the M_I_-M_III_ domain with weakened M_I_/M_II_–M_III_ interaction, we examined the MT M_I_-M_III_ fragment (force curves are shown in Supplementary Figure 3A). The contour map for MT M_I_-M_III_ showed two specific regions (*L*_c_ = 19.4 nm and 37.6 nm, arrowheads in Fig. 3A; the contour map with regard to net extension is shown in Supplementary Figure 3B). The regions had greater peak unfolding forces *F*_u_ (146.8 pN and 141.0 pN) than those for WT in loading (a) (Fig. 2B). Moreover, the number densities *n*_d_ in these regions were higher than those in WT (arrowheads, the side view of *n*_d_ distribution in Fig. 3B). Furthermore, in the later region (50 nm < *L*_c_ < 150 nm), two separate regions observed in WT M_I_-M_III_ (blue lines, upper part of Fig. 3B) fused into one broad region in MT (lower part of Fig. 3B). This observation indicated that M_II_–M_III_ domain changed its conformation because of weakened M_I_/M_II_–M_III_ interaction. The result for MT M_I_-M_III_ indicated that weakened M_I_/M_II_–M_III_ interaction triggers the changes in M_I_-M_III_ conformation.

Individual M_I_ and M_II_–M_III_ fragments showed one and two regions, respectively (Fig. 3C; force curves are shown in Supplementary Figure 3C,D) with higher *n*_d_ values (Supplementary Figure 3E) than those for M_I_-M_III_ fragments, indicating that M_I_ and M_II_–M_III_ domains settled into an innate stable state without interacting with each other. In particular, the peak unfolding force for the M_I_ domain (155.9 pN, Fig. 3C) was approximately 50% greater than that for the corresponding region of WT M_I_-M_III_ in loading (a) (107.7 pN, Fig. 2B). This result indicated that the weak helix bundle of M_I_ changed the conformation to another state with higher mechanical stability without the interaction with M_II_–M_III_ domain.

To compare the mechanical stabilities of α-catenin fragments, we analyzed the number of force peaks *N* per curve, as shown in Fig. 3D. The stacked bar graphs (Fig. 3D) display *N* values, with color intensity illustrating the range of transition force Δ*F*_T_ for force peaks (Fig. 1C). The greater Δ*F*_T_ values correspond to more stable substructures. For WT M_I_-M_III_ fragment (orange and purple bars, Fig. 3D), *N* values at large Δ*F*_T_ (>100 pN, associated with a stable substructure) increased in loading (b) compared with that in loading (a). On the other hand, the value of *N* at small Δ*F*_T_ (20 pN to 40 pN, associated with unstable substructure) decreased in loading (b). This result supported our idea that the conformation of WT M_I_-M_III_ changed to a more stable state with weakened M_I_/M_II_–M_III_ interaction under tension. In addition, the sum of the *N* values for M_I_ (green bar, Fig. 3D) and M_II_–M_III_ (blue bar) was greater than that for WT M_I_-M_III_ in loading (a). This result suggested that the M_I_/M_II_–M_III_ interaction destabilized the M_I_ and M_II_–M_III_ conformations under no force. On the basis of the results for mutated and segmented α-catenin fragments, we determined that the M_I_/M_II_–M_III_ interaction acted as an intramolecular switch to induce the mechano-adaptive conformational change of M_I_-M_III_ domain.

### Reinforcing vinculin binding to M_I_-M_III_

**Figure 4:**
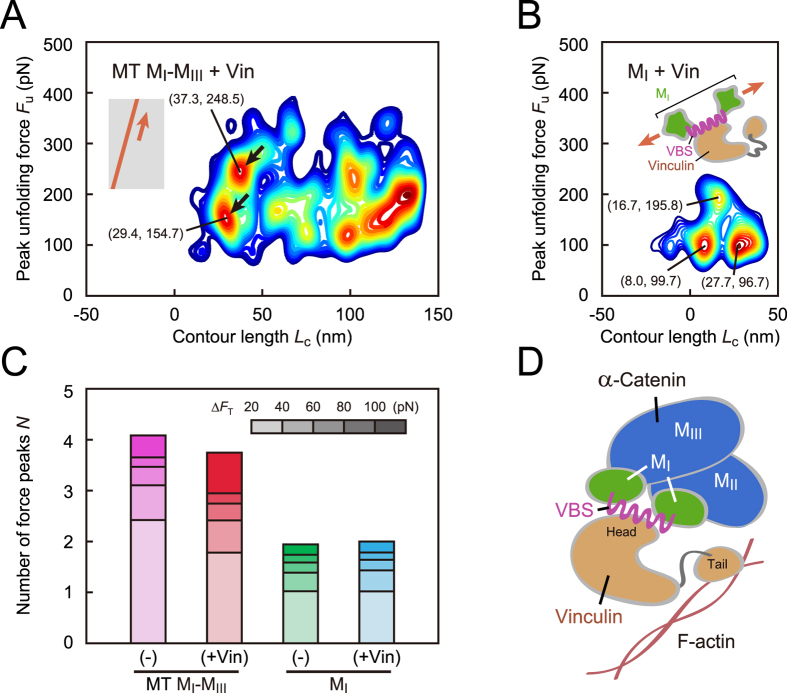
The effect of vinculin binding on mechanical behavior of α-catenin. (**A**) Contour map for vinculin-bound MT M_I_-M_III_ fragment based on 571 force curves. (**B**) Contour map for vinculin-bound M_I_ fragment based on 111 force curves. The conformation of vinculin-bound M_I_ domain comprised a stable helix of VBS with high *F*_u_ and two unstable substructures with low *F*_u_. (**C**) Changes in the average number of force peaks *N* caused by vinculin binding. (**D**) Schematic of the molecular complex consisting of α-catenin, vinculin, and actin filament (F-actin). M_I_-M_III_ domain was reinforced by vinculin binding at the head domain, in which the M_II_–M_III_ domain stabilized the conformation of vinculin-bound M_I_ domain. The tail domain of vinculin associates with another actin filament.

To elucidate how the vinculin binding affects the mechanical behavior of α-catenin, we examined the α-catenin fragments after vinculin treatment. Vinculin binds to an α-catenin molecule with the head domain, and the tail domain associates with another actin filament. The contour map of *n*_d_ for vinculin-bound MT M_I_-M_III_ showed characteristic regions (*L*_c_ = 29.4 nm and 37.3 nm; arrows in Fig. 4A; force curves are shown in Supplementary Figure 4A) at a greater peak unfolding force *F*_u_ (154.7 pN and 248.5 pN) than that for MT M_I_-M_III_ without vinculin (Fig. 3A). This result indicated that M_I_-M_III_ domain was reinforced by vinculin binding. In contrast to MT M_I_-M_III_, vinculin-bound M_I_ (Fig. 4B; force curves are shown in Supplementary Figure 4B) showed two regions at smaller *F*_u_ (99.7 pN and 96.7 pN) than M_I_ without vinculin (155.9 pN, Fig. 3C). Vinculin-bound M_I_ showed one region at a rather greater *F*_u_ (195.8 pN, Fig. 4B). This result indicated that the conformation of vinculin-bound M_I_ comprised a stable helix of VBS and two unstable substructures remaining in this domain.

Comparing the results for MT M_I_-M_III_ and M_I_ fragments, we concluded that the M_II_–M_III_ domain stabilized the unstable vinculin-bound M_I_ domain. Thus, the number of force peaks *N* at large Δ*F*_T_ (>100 pN) for MT M_I_-M_III_ was increased by vinculin binding (red bar, Fig. 4C), with a decrease in *N* at small Δ*F*_T_ (20 pN–40 pN). The number of force peaks for M_I_ did not change significantly (cyan bar, Fig. 4C). The stabilizing role of M_II_–M_III_ domain was further confirmed in the analysis of total energy *E*_tot_ for completely unfolding (Supplementary Figure 5). *E*_tot_ for M_I_ decreased after vinculin binding (cyan bar) that was caused by greater decrease in unfolding energy for two unstable substructures than the increase in unfolding energy for stable VBS helix. However, MT M_I_-M_III_ increased after vinculin binding (red bar). The results for vinculin-bound α-catenin fragments revealed that M_I_-M_III_ domain was reinforced by vinculin binding at the head domain (Fig. 4D), with M_II_–M_III_ domain stabilizing the conformation of the vinculin-bound M_I_.

### Changes in the persistence length of M_I_-M_III_ as a polymer chain

**Figure 5:**
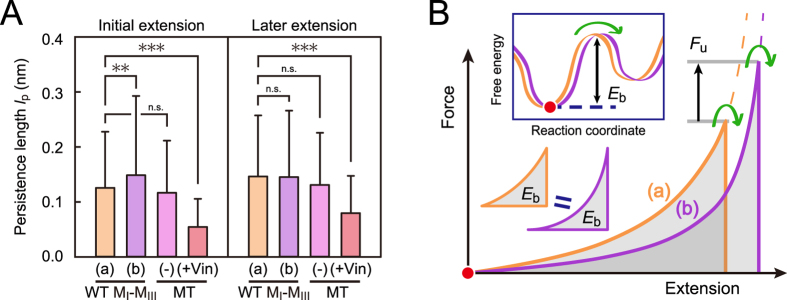
Changes in the persistence length of α-catenin as a polymer chain. (**A**) Comparison of persistence lengths *l*_p_ in M_I_-M_III_ fragments. Statistical significance of the differences was analyzed using *t*-test (**p < 0.01 and ***p < 0.005). The error bars show standard deviations (S.D.). Persistence length *l*_p_ was analyzed in the initial extension (0 nm < *L*_c_ ≤ 72 nm) and the later extension (72 nm < *L*_c_ < 144 nm). (**B**) The effect of persistence length on the peak unfolding force. If we postulate the same energy barrier *E*_b_ for the next intermediate state in M_I_-M_III_ before and after the activation (orange (a) and purple (b) lines), greater peak unfolding force *F*_u_ will be required to overcome the *E*_b_ in the activated M_I_-M_III_ (b), with greater *l*_p_, than in M_I_-M_III_ (a).

To examine the changes in the α-catenin molecule as a polymer chain, we analyzed the persistence length *l*_p_ (Fig. 5A). In the initial extension (0 nm < *L*_c_ ≤ 72 nm, the left plot in Fig. 5A), the persistence length *l*_p_ for WT M_I_-M_III_ in loading (b) (purple bar) was greater than in loading (a) (orange bar). This result indicated that the persistence length was increased by mechanical activation, resulting in the decreased tensile force required to a certain amount of extension (Fig. 5B). No significant differences were observed in the later extension (72 nm < *L*_c_ < 144 nm, the right plot in Fig. 5A). After vinculin binding, *l*_p_ settled to a much smaller value in the entire extension (red bars in Fig. 5A), indicating that the M_I_-M_III_ domain was immobilized because of the conformational reinforcement caused by vinculin.

Here, the increase in *l*_p_ of M_I_-M_III_ by mechanical activation was consistent with the increase in the peak unfolding force *F*_u_. We can postulate the same energy barrier *E*_b_ for the next intermediate state in M_I_-M_III_ before and after the activation (Fig. 5B) on the basis of the total unfolding energy *E*_tot_ (Supplementary Figure 5). Further, the activated M_I_-M_III_ (b), with greater *l*_p_, should be extended more to overcome the energy barrier *E*_b_ than M_I_-M_III_ (a), with smaller *l*_p_, resulting in a greater peak unfolding force *F*_u_. Thus, the M_I_-M_III_ domain of α-catenin under tension changes the conformation to an intermediate state with a larger persistence length, and finally settles into the immobilized state caused by the reinforcement with vinculin.

## Discussion

On the basis of our single-molecule experiments, we revealed the mechano-adaptive sensory mechanism of α-catenin. Under physiologically possible low tension, α-catenin adaptively changed the conformation to a stable intermediate state. Such mechano-adaptive conformational changes enable α-catenin at AJs to retain the activated state under tension, without successive unfolding, to function as a robust tension sensor. Our findings could be one solution to a paradox. The tension-sensing proteins require mechanical forces for their activation; however, they have to be stable under such conditions because the mechanical forces function as basic protein denaturants. Furthermore, we revealed that vinculin-binding reinforces α-catenin conformation; the stable α-catenin-vinculin complex contributes to the tight anchoring of adhesive molecules at AJs^29^ by recruiting another actin filament. Therefore, we suggest that the mechano-adaptive sensory ability of α-catenin arises from its molecular plasticity in response to both mechanical and biochemical cues.

Our results for mutated (MT M_I_-M_III_) and segmented (M_I_, M_II_–M_III_) α-catenin reveal that the M_I_/M_II_–M_III_ interaction acts as a conformational switch to the intermediate state, where M_I_ and M_II_–M_III_ domains change to the conformations with increased stability. Our results are consistent with the previous reports that the helix bundle of M_I_ requires the structural stabilization by M_II_–M_III_^28^ and that helix bundles M_II_–M_III_ are approximately 180°-rotated in conformation without M_I_ domain^30^,^31^. However, there were some discrepancies between the results for MT M_I_-M_III_ without mechanical activation and those for WT M_I_-M_III_ in loading (b) with mechanical activation. Thus, we should consider both the importance of tension for the drastic conformational changes and the conformational switch caused by M_I_/M_II_–M_III_ interaction.

In our experiments, the full-length vinculin firmly bound to MT M_I_-M_III_ fragment, with an increase in peak unfolding force *F*_u_ and a decrease in the persistence length *l*_p_. The full-length vinculin assumes an autoinhibited conformation for α-catenin-binding^32^. Our results suggested that the VBS exposed in the mechanically-activated α-catenin opens the autoinhibited conformation of vinculin to make a stable α-catenin–vinculin complex. Our data supported the previous reports^24^,^33^ that α-catenin and vinculin are “co-activated” for interacting with each other under tension. This type of a force-induced vinculin-activation mechanism of α-catenin could be conserved in talin and α-actinin that are similar adhesive proteins constituting a molecular complex with vinculin and actin filament^34^.

The loading rate used here was appropriate for the qualitative analysis of the mechanical behavior of α-catenin. Our results for WT M_I_-M_III_ in loading (a) were in agreement with the results of the experiments (performed using magnetic tweezers) using a low loading rate (approximately 4 pN/s)^18^. Moreover, the “holding” method utilized in our study allowed us to analyze the conformational changes under physiologically possible low tension of approximately 10 pN. Thus, by introducing the holding time into fast loadings, we succeeded in analyzing the conformational changes of α-catenin molecules under low tension while efficiently exploring their unfolding trajectories.

The persistence length *L*_p_ appeared in this study was smaller than that reported (approximately 0.4 nm) for titin molecules^35^. This discrepancy may be related to the secondary structures of molecules. A previous study^36^ employing molecular dynamics simulation revealed that a protein with high φ dihedral potential shows a small persistence length of 0.19 nm. This suggests that α-helical proteins such as α-catenin, of which φ dihedral angle is more constrained than β-sheet proteins such as titin, exhibit smaller persistence length. In addition, subcomponents in α-catenin molecules, such as helix bundles, could decrease the persistence length *L*_p_.

Based on our study, we propose a novel concept of “mechano-adaptive” molecules that fulfill their innate functions by adapting to the cellular forces. Under these forces, biomolecules such as proteins and nucleic acids may change their conformations, mechanical behaviors, and chemical properties. Such molecular-scale changes under mechanical forces could affect force-induced phenomena at cellular and tissue level. We believe that our study may be the basis for future studies investigating the concept of mechano-adaptive sensory mechanism.

## Methods

### Protein purification

DNA fragments of mouse WT αE-catenin M_I_-M_III_, MT M_I_-M_III_, M_I_, and M_II_–M_III_ were amplified by PCR and cloned into the pGEX6P-3 vector (GE Healthcare). All plasmids were verified by DNA sequencing and transformed into *Escherichia coli* strain BL21Star (DE3) (Invitrogen) cells for protein expression. Protein expression was performed at 20 °C in Luria–Bertani medium containing 0.1 mM isopropyl-β-D-thiogalactopyranoside. Cells expressing αE-catenin were suspended in 20 mM Tris-HCl buffer (pH 8.0) containing 150 mM NaCl and disrupted by sonication. After ultracentrifugation, the supernatant was applied onto a Glutathione Sepharose 4B column (GE Healthcare). Eluted proteins were further purified by anion-exchange (HiTrap Q HP, GE Healthcare) and gel filtration (Superdex 200 pg, GE Healthcare) chromatography.

### Chemical modification

For SMFS, a glass substrate and AFM tip were treated using a chemical modification process^21^. The glass substrate was modified with α-catenin at its C-terminus and AFM tip was modified with glutathione, which interacts with N-terminal GST-tag of α-catenin. The glass substrate was oxidized and treated with 2% MPTMS/ethanol for 15 min. The substrate was then treated with 2 mM maleimide-C_3_-NTA (Mal-C_3_-NTA; DOJINDO Lab.)/PBS for 30 min, with 10 mM NiCl_2_ (Wako Pure Chemical Industries)/Milli-Q for 30 min, and washed with PBS. α-Catenin fragments (10 μM for each fragment) were modified by NTA-Ni^2+^-His_6_ affinity binding for 1 h and finally washed with working buffer (10 mM HEPES, 150 mM NaCl, pH 7.2). For the SMFS of vinculin-bound α-catenin fragments, the α-catenin-modified substrate was further incubated with 1 μM full-length vinculin/PBS for 30 min and washed with working buffer. Silicon nitride AFM tip (OMCL-TR400PSA-1; spring constant, 0.02 N/m, Olympus Co.) was first oxidized using ozone cleaner and treated with 2% APTES/ethanol for 15 min. The tip was then treated with 1.5 mM Mal-PEG-NHS ester/PBS for 30 min and with 10 mM glutathione/PBS for 1 h. The remaining maleimide was quenched with 50 mM 2-mercaptoethanol/HEPES and finally washed with working buffer.

### Force curve analysis

Force curves with saw-tooth peaks, caused by conformational transitions of α-catenin, were analyzed using the in-house software (Fig. 1C). First, we extracted the force curves for completely extended single α-catenin molecules, based on the thresholds of force and stiffness at the rupture event, assuming an 85%-extended worm-like chain model^37^,


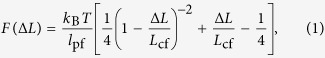


where *k*_B_ is the Boltzmann constant, *T* is temperature [300 K], *l*_pf_ is the final persistence length [0.4 nm] based on a previous report^35^. The final contour length *L*_cf_ was estimated as 143.2 nm (WT/MT M_I_-M_III_), 46.8 nm (M_I_), and 99.6 nm (M_II_–M_III_) based on the number of residues. The threshold of the stiffness excluded the curves with low stiffness for aggregated molecules. For the extracted curves, we determined the length of offset *L*_offset_, corresponding to PEG length (approximately 45 nm) and the tip curvature radius, by fitting WLC model allowing baseline offset as


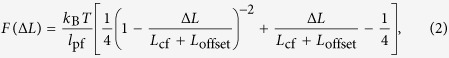


to the final peak at the rupture event (green line, Fig. 1C), where *L*_offset_ was a fitting parameter. Finally, we identified the intermediate force peaks with the transition force Δ*F*_T_ (cyan arrow, Fig. 1C) based on the threshold Δ*F*_th_ (40 pN) and calculated the contour length *L*_c_ and the persistence length *l*_p_ by fitting WLC model with the determined *L*_offset_ to the force peaks as


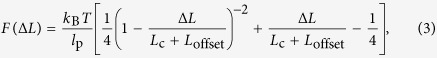


by considering that the PEG linker, without any substructures, can be fully unfolded at each intermediate state. To analyze the mechanical stabilities for intermediate states, we measured peak unfolding force *F*_u_ (magenta arrow, Fig. 1C). We adopted lower Δ*F*_th_ (20 pN) to detect the force peaks caused by unfolding of unstable substructures.

### Number density of force peaks

Based on scatter plots of peak unfolding force *F*_u_ versus contour length *L*_c_, we evaluated the number density *n*_d_ (*L*_c_, *F*_u_) of force peaks per curve by taking the product of the average number of force peaks per one curve *N* and the probability density *P* (*L*_c_, *F*_u_) calculated using two-variable Gaussian distribution function as


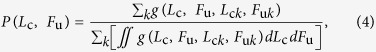


where


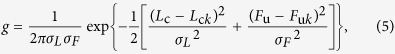


in which the summation for *k* meant the summation for all scattered data points.

## Additional Information

**How to cite this article**: Maki, K. *et al.* Mechano-adaptive sensory mechanism of α-catenin under tension. *Sci. Rep.* **6**, 24878; doi: 10.1038/srep24878 (2016).

## Supplementary Material

Supplementary Information
