# Supplementary material for: Mechano-adaptive sensory mechanism of α-catenin under tension
Source: Sci Rep. 2016 Apr 25;6:24878. doi: 10.1038/srep24878 (PMC4843013; doi:10.1038/srep24878)
Supplement: Supplementary Information [file srep24878-s1.pdf]

Supplementary Information

Mechano-adaptive sensory mechanism of α-catenin under tension

Koichiro Maki[1,2], Sung-Woong Han[3], Yoshinori Hirano[4], Shigenobu Yonemura[5], Toshio Hakoshima[4], Taiji Adachi[1,2]

[1]Department of Biomechanics, Institute for Frontier Medical Sciences, Kyoto University, 53 Shogoin-Kawahara-cho, Sakyo, Kyoto 606-8507, Japan

[2]Department of Micro Engineering, Graduate School of Engineering, Kyoto University, Yoshida Honmachi, Sakyo, Kyoto 606-8501, Japan

[3]National Institute for Nanomaterials Technology, Pohang University of Science and Technology, 77 Cheongam-ro, Nam-Gu, Pohang, Gyeongbuk 790-784, Korea

[4]Structural Biology Laboratory, Graduate School of Biological Sciences, Nara Institute of Science and Technology, 8916-5 Takayama, Ikoma, Nara 630-0192, Japan

[5]Ultrastructural Research Team, RIKEN Center for Life Science Technologies, 2-2-3 Minatojima-minamimachi, Chuo-ku, Kobe, Hyogo 650-0047, Japan

*Corresponding author:

T. Adachi, Department of Biomechanics, Institute for Frontier Medical Sciences, Kyoto University, 53 Shogoin-Kawahara-cho, Sakyo, Kyoto 606-8507, Japan

Tel.: +81-75-751-4853; Fax: +81-75-751-4853; E-mail: adachi@frontier.kyoto-u.ac.jp

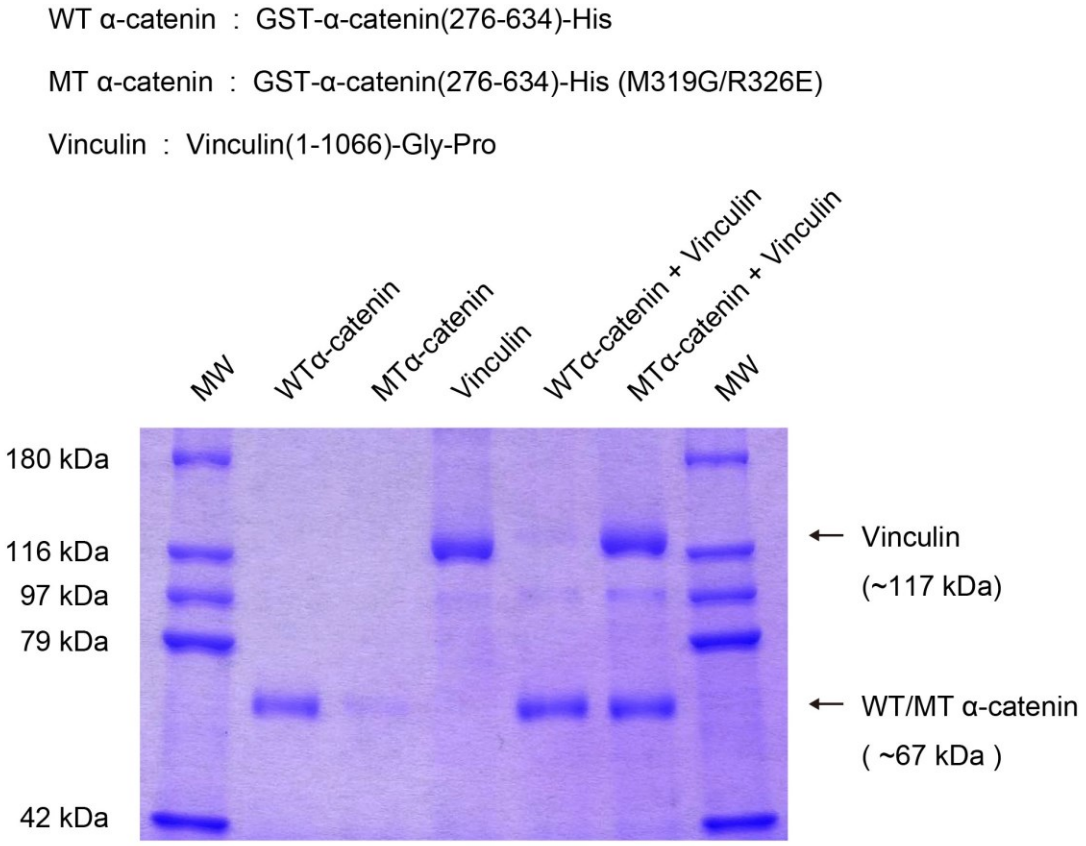

Fig. S1. Pull-down assay for α-catenin samples with vinculin. WT and MT (M319G and R326E) α-catenin M_I-M_{III} fragments and full-length vinculin were mixed and incubated in solution and were applied to GST SpinTrap (GE Healthcare). After two times washing, α-catenin and vinculin were eluted together. Autoinhibitory WT α-catenin did not show vinculin affinity. In contrast, vinculin was bound to MT α-catenin, indicating that the mutations disrupted the autoinhibiting M_I/M_{II}-M_{III} interaction while conserving the vinculin binding affinity.

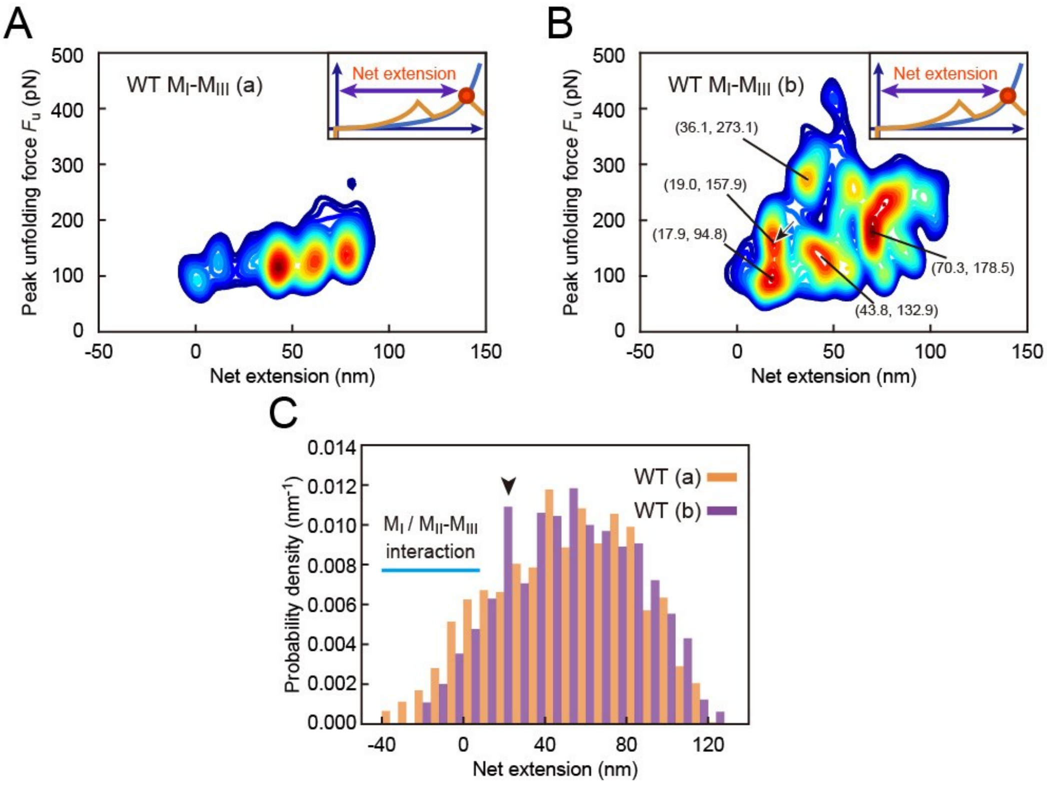

Fig. S2. Analysis of the net extension of force peaks. (A) Contour map of number density n_d of force peaks for WT M_I-M_{III} in loading (a) against the net extension. (B) Contour map in loading (b) against the net extension. The initial peak (arrow) corresponded to the specific peak indicated in Fig. 2D. (C) Histograms of the net extension of WT M_I-M_{III} in loading (a) (orange bars) and loading (b) (purple bars). The arrowhead shows the salient peak in loading (b) corresponding to the peak indicated in the contour map. In the initial extension, the probability density in loading (b) was lower than that in loading (a), indicating that the M_I/M_{II}-M_{III} interaction was partly diminished during the holding time in loading (b).

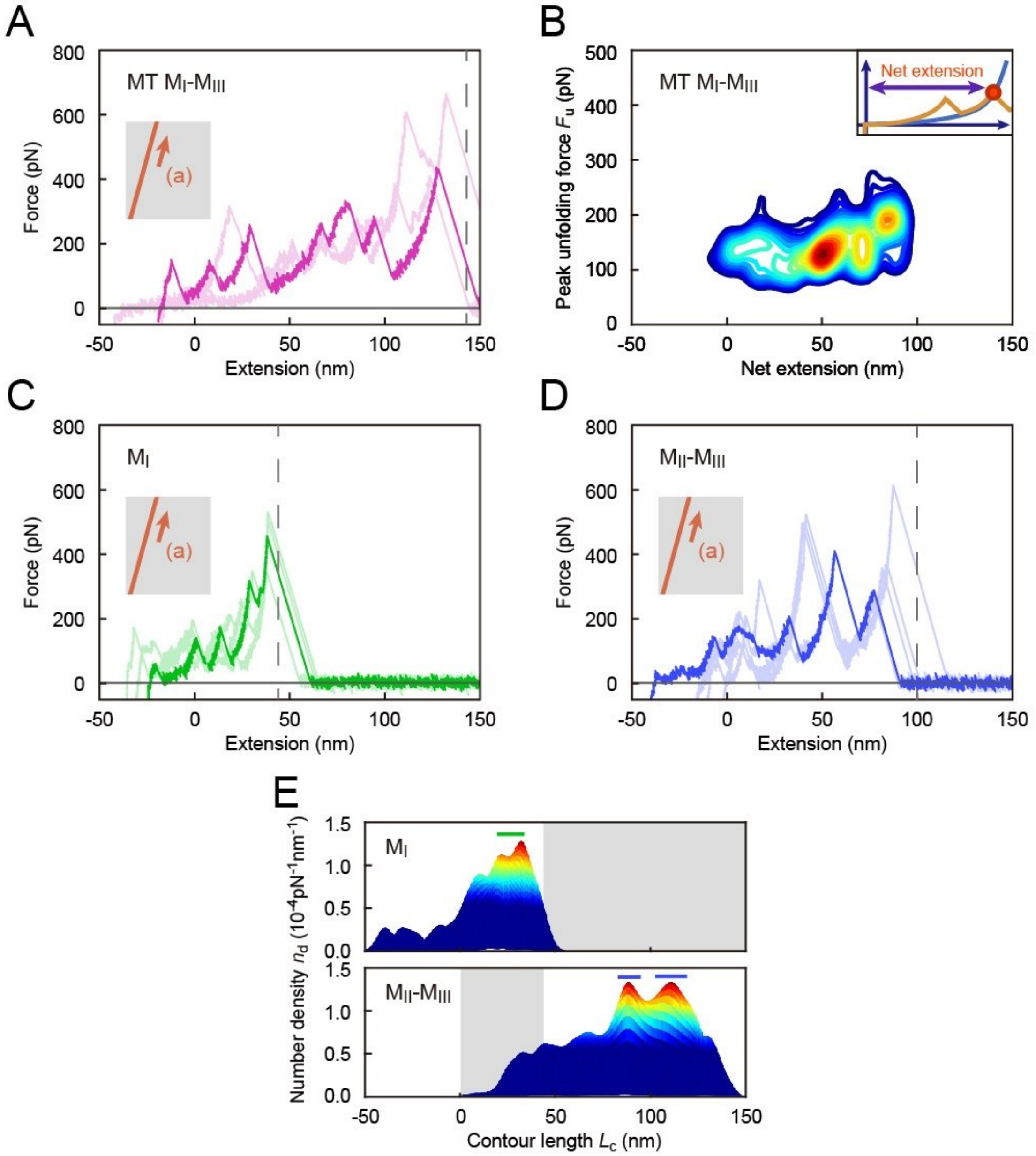

Fig. S3. Force curves and number density n_d for mutated and segmented fragments.

(A) Force curves for MT M_I-M_{III}. (B) Contour map for MT M_I-M_{III} against the net extension. (C, D) Force curves for M_I and M_{II}-M_{III}. (E) Comparison of the number density n_d in contour maps against contour length L_c from a side view in M_I and M_{II}-M_{III} fragments. The lower part of M_{II}-M_{III} is shifted to the right by 46.8 nm that is the fully-extended length of M_I domain.

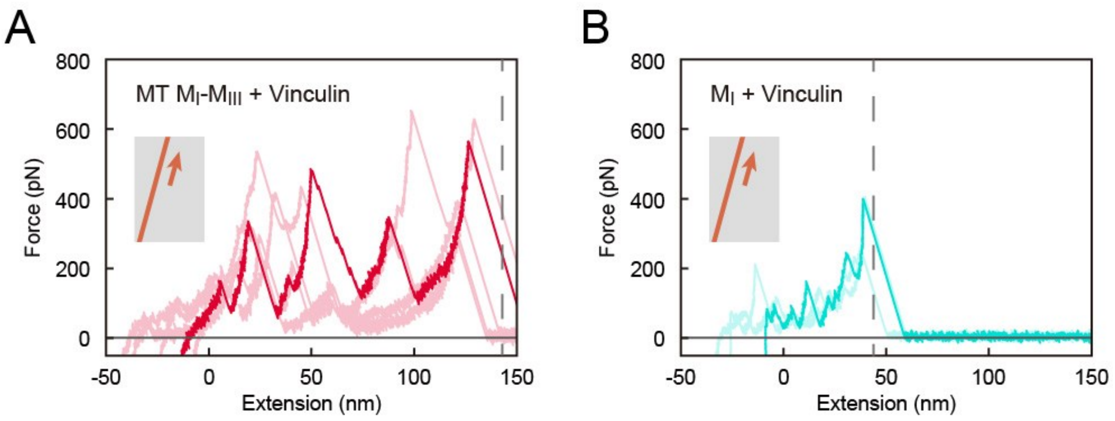

Fig. S4. Force curves for vinculin-bound MT M_I-M_{III} and M_I fragments. (A) The vinculin-bound MT M_I-M_{III} fragment showed greater peak unfolding force F_u than the fragment without vinculin. (B) F_u of M_I fragment was decreased by vinculin binding.

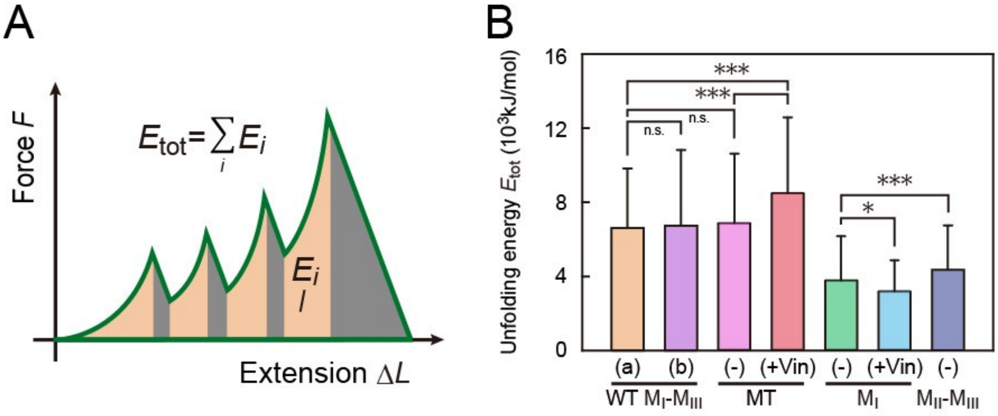

Fig. S5. Total unfolding energy for examined fragments. (A) Schematic of the analysis of the total unfolding energy E_{tot}. The total unfolding energy E_{tot} (orange area) was calculated by subtracting the cantilever bending energy (gray area) from the piezo-moving energy (green-enclosed area). (B) In the bar chart of E_{tot} for examined fragments, the statistical significance of the differences was analyzed using *t*-test (*, $p < 0.05$ and ***, $p < 0.005$). E_{tot} for MT M_I-M_{III} fragment significantly was increased by vinculin binding, while no significant differences were observed for WT and MT M_I-M_{III} fragments without vinculin binding.
